# Regulatory Diversity and Functional Analysis of Two-Component Systems in Cyanobacterium *Synechocystis* sp. PCC 6803 by GC-MS Based Metabolomics

**DOI:** 10.3389/fmicb.2020.00403

**Published:** 2020-03-17

**Authors:** Mengliang Shi, Lei Chen, Weiwen Zhang

**Affiliations:** ^1^Laboratory of Synthetic Microbiology, School of Chemical Engineering & Technology, Tianjin University, Tianjin, China; ^2^Frontier Science Center for Synthetic Biology and Key Laboratory of Systems Bioengineering, Ministry of Education of China, Tianjin, China; ^3^Collaborative Innovation Center of Chemical Science and Engineering, Tianjin, China; ^4^Center for Biosafety Research and Strategy, Tianjin University, Tianjin, China

**Keywords:** *Synechocystis*, GC-MS, metabolomics, two-component signal transduction systems, response regulators

## Abstract

Two-component signal transduction systems are still poorly functionally characterized in the model cyanobacterium *Synechocystis* sp. PCC 6803. To address the issue, a GC-MS based comparative metabolomic analysis was conducted on a library of 44 knockout mutants for the response regulators (RRs) in *Synechocystis*. The metabolomic profiling analysis showed that 7 RRs mutants, namely Δ*slr1909*, Δ*sll1291*, Δ*slr6040*, Δ*sll1330*, Δ*slr2024*, Δ*slr1584*, and Δ*slr1693*, were significantly different at metabolomic level, although their growth patterns are similar to the wild type under the normal autotrophic growth condition, suggesting regulatory diversity of RRs at metabolite level in *Synechocystis*. Additionally, a detailed metabolomic analysis coupled with RT-PCR verification led to useful clues for possible function of these 7 RRs, which were found involved in regulation of multiple aspects of cellular metabolisms in *Synechocystis*. Moreover, an integrative metabolomic and evolutionary analysis of all RR showed that four groups of RR genes clustered together in both metabolomic and evolutionary trees, suggesting of possible functional conservation of these RRs during the evolutionary process. Meanwhile, six groups of RRs with close evolutionary origin were found with different metabolomic profiles, suggesting possible functional changes during evolution. In contrast, more than 10 groups of RR genes with different clustering patterns in the evolutionary tree were found clustered together in metabolomics-based tree, suggesting possible functional convergences during the evolution. This study provided a metabolomic view of RR function, and the most needed functional clues for further characterization of these regulatory proteins in *Synechocystis*.

## Introduction

Cyanobacteria, as one of the primary producers for life on earth, have attracted significant attention in recent decades due to its ability to grow oxygenic photo-autotrophically. In nature, cyanobacteria are found in varying environments ranging from frigidity and torridity, alkaline and acid, saline ocean and freshwater, terrestrial, and symbiotic environments (Ashby and Houmard, 2006), probably due to their abilities to adjust cell behavior by regulating gene expression accurately and timely to adapt to various environmental perturbations. Cellular responses of cyanobacteria to various environmental factors, such as oxidative stress (Cassier-Chauvat and Chauvat, 2014), osmotic stress (Ai et al., 2014), salt (Pade and Hagemann, 2015), phosphate (Schwarz and Forchhammer, 2005), heavy metal ion (Cassier-Chauvat and Chauvat, 2014), acids (Hirani et al., 2001), nitrogen starvation (Schwarz and Forchhammer, 2005), cold stress (Sinetova and Los, 2016), heat shock (Rajaram et al., 2014), and high light stress (Muramatsu and Hihara, 2012), have been a research focus for many years. Additionally, due to its readiness for gene manipulation (Shestakov and Khyen, 1970), cyanobacteria have been recently developed as a “chassis” in synthetic biology to produce multiple biofuels and bioproducts, namely, 3-hydroxypropionic acid (Wang et al., 2016), free fatty acids (Liu et al., 2011), isoprene (Chaves and Melis, 2018), 2,3-butanediol (Oliver et al., 2013), 1-butanol (Lan and Liao, 2011), squalene (Englund et al., 2014), and sunscreen compound mycosporin-like amino acids (Singh et al., 2017), demonstrating their feasibility as a “microbial cell factories” for future large-scale biotechnology application.

As one of the critical signal transduction systems in microbes, the two-component signal transduction systems (TCSTSs) are typically composed of a histidine kinase (HK) as a transmitter sensor and a response regulator (RR) as a receiver, controlling the gene expression of microbes in response to the alterations in environments (Ashby and Houmard, 2006). HK responds to environmental signals and then its conserved histidine residue is auto-phosphorylated. The autophosphorylation then enables HK to bind and convey its specific phosphoryl group onto a conserved aspartate residue on a RR. Subsequently, the RR is phosphorylated on its conserved receiver domain, which normally results in the activation of an output domain that can alternate the expression activities in a variety of genes that allow the microbes to adapt to the external signals (Laub et al., 2007). Among the 3,725 putative genes in *Synechocystis* sp. PCC 6803 (hereafter *Synechocystis*), 45 genes on chromosome and plasmids encode putative RRs (Kaneko et al., 1996; Ashby and Houmard, 2006). Several RRs have been functionally characterized, such as Slr1909 mediating acid response network (Ren et al., 2014) and Sll0649 involved in tolerance to cadmium and several other metal ions (Chen et al., 2014b), as well as Sll0797 (*rppA*) and Sll0798 (*rppB*) controlling the expression of genes involved photosystem I and II (Li and Sherman, 2000), Slr0115, Slr0649, Sll1708, and Slr1783 involved in salt and hyperosmotic stress (Shoumskaya et al., 2005), RpaA involved in adaption to changes in light conditions (Iijima et al., 2015), RpaB repressing high light inducible genes (Kappell and Van Waasbergen, 2007), and activating PSI under low light condition (Seino et al., 2009), and Slr0115 (RpaA) and Slr0947 (RpaB) oppositely controlling the flow of energy from the light-harvesting phycobilisomes to the photosystems (Kappell and Van Waasbergen, 2007). In addition, a previous work by Murata and Suzuki indicated that multiple sets of Hik-Rre pairs work for transcriptional regulation of different sets of genes under different conditions (Murata and Suzuki, 2006), for example, Hik33-Rre26/Rre31 was identified to respond to low-temperature stress, Hik33/Hik34-Rre31, Hik10-Rre3, Hik16/Hik41-Rre17, Hik2-Rre1 were reported to respond to salt and hyperosmotic stress (Murata and Suzuki, 2006). However, in spite of all the progress, most of the RRs in *Synechocystis* are still functionally unknown.

Various genome-scale methodologies have recently been developed and utilized to explore gene function of TCSTSs in a variety of microbes. For example, a genome-scale transcriptomic analysis in the parental *Streptomyces coelicolor* strain M145 and the mutant Δ*draR*-*K* was conducted via DNA microarray and the real-time reverse transcription quantitative polymerase chain reaction (RT-qPCR), unveiling the global function of DraR-K in regulating the differentiation of physiology and morphology (Yu et al., 2014). In our previous work, the possible connection between the small RNA CoaR and the RR Slr1037 in regulating tolerance to butanol in *Synechocystis* was identified and confirmed separately through quantitative proteomics and quantitative real-time PCR (qRT-PCR) (Sun et al., 2017a). Metabolomics can identify numerous small molecules in cell and show their variances in abundance, which allows it to be extensively utilized in the analysis of various microbes in responsive to alterations in environmental conditions. In a recent study, a targeted liquid chromatography-mass spectrometry (LC-MS) based metabolomic profile analysis showed a widespread crosstalk of TCSTSs in regulating central and energetic metabolism in *Synechocystis* (Pei et al., 2017). However, among the limited metabolites selected in this targeted LC-MS based metabolomic profile analysis, most of the metabolites were unstable in room temperature (Pei et al., 2017). Compared with LC-MS based approach, untargeted gas chromatography-mass spectrometry (GC-MS) based metabolomic analysis can detect more metabolites, which include some stable small molecules such as various organic acids, amino acids and sugars, thus provide more information on the metabolism regulation of TCSTSs. In this study, to further elucidate the functions of TCSTSs in *Synechocystis*, a GC-MS based metabolomic profiling was conducted for 44 knockout mutants of *Synechocystis* RR-coding genes under photoautotrophic growth condition. The metabolomic analysis showed that seven mutants have the greatest changes at the metabolite level compared with the wild type, indicating their differential regulatory roles. Additionally, an integrative analysis of metabolomic profiles and evolutionary trees was also conducted to reveal the functional conservation and changes of RRs during evolution process in *Synechocystis*.

## Materials and Methods

### Bacterial Growth Conditions

*Synechocystis* sp. PCC 6803 was attained from American Type Culture Collection (ATCC) and utilized as the wild type to construct single-gene knockout mutants of RR genes. A total of 44 knockout mutants of putative RR-coding genes were successfully constructed, confirmed and described previously (Pei et al., 2017). The mutants and the wild type grew in the normal BG11 medium (pH 7.5) in 100 mL flasks with 25 mL medium in each flask. The light intensity was generally 50 μmol photons m^−2^s^−1^ and the illumination incubator was set at 130 rpm, 30°C (HNY-211B Illuminating Shaker, Honour, China). The condition of growth was defined as the cell density measured at OD_730nm_ by a UV-1750 spectrophotometer (Shimadzu, Japan) every 12 h, from the starting point at about OD_730nm_ 0.1 and after 48 h at ~OD_730nm_ 1.25. For each strain, five biological replicates were inoculated independently, and the samples were collected for the subsequent metabolomic analysis.

### GC-MS Based Metabolomic Analysis

All chemicals used for metabolites isolation and GC-MS analysis were accessed from Sigma-Aldrich (Taufkirchen, Germany). For metabolomic analysis, samples of the wild type and the mutants were collected from normal BG11 medium at 48 h. For each sample, cells equivalent to 10^8^ were collected by centrifugation at 6,000 × g for 10 min at 4°C (Eppendorf, Hamburg, Germany). The cell pellets were immediately frozen in liquid nitrogen and then stored at −80°C before use. The metabolomic analysis was performed as described previously (Zhang et al., 2015). Peak areas of all the metabolites identified in GC-MS were normalized by the peak areas of internal standard D^13^-sorbitol and the cell numbers of the sample, and the comparative abundances for each identified metabolite were used for future analysis. The metabolites missing in more than 50% of the samples were cut off and <50% were filled up with average in replicates.

### Statistical Analysis for Metabolomic Data

The metabolomic profiles were then further standardized via dividing the relative values of mutants by that of the wild type, and taking log_2_ of the results. The data were applied to Principal Component Analysis (PCA) via the software SIMCA-P 11.5 (Laiakis et al., 2010). Samples with *p* < 0.05 displayed by hoteling *t*^2^ of were considered distinctly different.

### qRT-PCR Analysis

Samples of *Synechocystis* mutants and the wild type were collected at 48 h. About 2 mL of samples with cell density of OD_730__nm_ = 2 were collected by centrifugation (12,000 × g for 2 min), and immediately applied to RNA extraction procedure following the manufacturer's protocol. Total RNA extraction was achieved through a Direct-zol™ RNA MiniPrep Kit (Zymo, CA, USA). cDNAs were synthesized via RevertAid First Strand cDNA Synthesis Kit as instructed in the manufacturer's protocol (Thermo Fisher Scientific Inc., CA, USA). Then the cDNAs for each sample was diluted for 500 times and 1 μL of each was employed as the template for the subsequent qRT-PCR reaction. All the primers were designed via primer-blast in the NCBI (http://www.ncbi.nlm.nih.gov) and synthesized by GENEWIZ Inc. (Suzhou, China). Primers used in this study are listed in Table S1. The qPCR reaction was carried out in 10 μL reactions composed of 5 μL of UltraSYBR Mixture (CW Biotech, Beijing, China), 3 μL dH_2_O, 1 μL diluted template cDNA, and 1 μL mix of each former and reverse PCR primer diluted by 500 times, utilizing the StepOne™ Real-Time PCR System (Applied Biosystems, CA, USA) (Sun et al., 2017b). Four technical replicates were performed for each condition. Data analysis was carried out using the StepOne analytical software v2.3 (Applied Biosystems, CA, USA) and the 2^−ΔΔCT^ method (Livak and Schmittgen, 2001). 16S rRNA was selected as a reference gene and the data were presented as ratios between the amounts of transcript in mutants and the wild type (Sun et al., 2017b).

### Comparative Analysis of Metabolomic and Evolutionary Profiles

Hierarchical clustering analysis based on the metabolomic profiles between each mutant and wild type, was performed. The metabolomic data of each mutant was normalized by that of the wild type in the same batch to avoid systematic error from the cultivation. Heatmap were created by MultiExperiment Viewer software available publically (http://mev.tm4.org/) (Howe et al., 2010). Then, protein sequences and receiver domain sequences of all the 44 response regulator genes were abstracted from NCBI (http://www.ncbi.nlm.nih.gov) and SMART (http://smart.embl-heidelberg.de/) (Letunic and Bork, 2017), then aligned by MUSCLE (http://www.ebi.ac.uk/Tools/msa/muscle/). To identify possible RR subfamilies, BLAST for homology identification was employed, and only those RR with 80% aligned coverage with *E*-value < 1e-20 were considered as the same families. Phylogenetic tree based on the conserved receiver domain of 44 RR genes was constructed by maximum likelihood method using the software Mega-X (Kumar et al., 2018) with 1,000 bootstrap replicates.

## Results and Discussion

### Untargeted GC-MS Based Metabolomic Analysis of RRs

In the previous study, attempt was made to generate single gene deletion mutants for all 45 tentative RR genes in the *Synechocystis* genome, and the efforts led to successful completely segregated construction of 41 RR genes and 3 partially knockout mutants *sll1879, sll0921, slr0947*, with only one, *slr6001* gene that we were not able to generate deletion mutant even after multiple attempts, implying the gene may be involved in essential cellular function under autotrophic growth condition. The growth patterns of 44 knockout mutants for RR genes in *Synechocystis* were compared with the wild type, and a targeted LC-MS based metabolomic analysis was conducted for their involvement in carbon and energetic metabolism under autotrophic or photomixotrophic conditions (Pei et al., 2017), where 24 chemically unstable key metabolites relative to central and energetic metabolism were selected and analyzed. However, the coverage of metabolites was significantly limiting toward metabolites in the central and energetic metabolism due to the limitation of targeted LC-MS approach. In this study, to further explore the possible functions of RRs in the metabolism regulation of *Synechocystis*, an untargeted GC-MS based metabolomic analysis was conducted.

**Figure 1:**
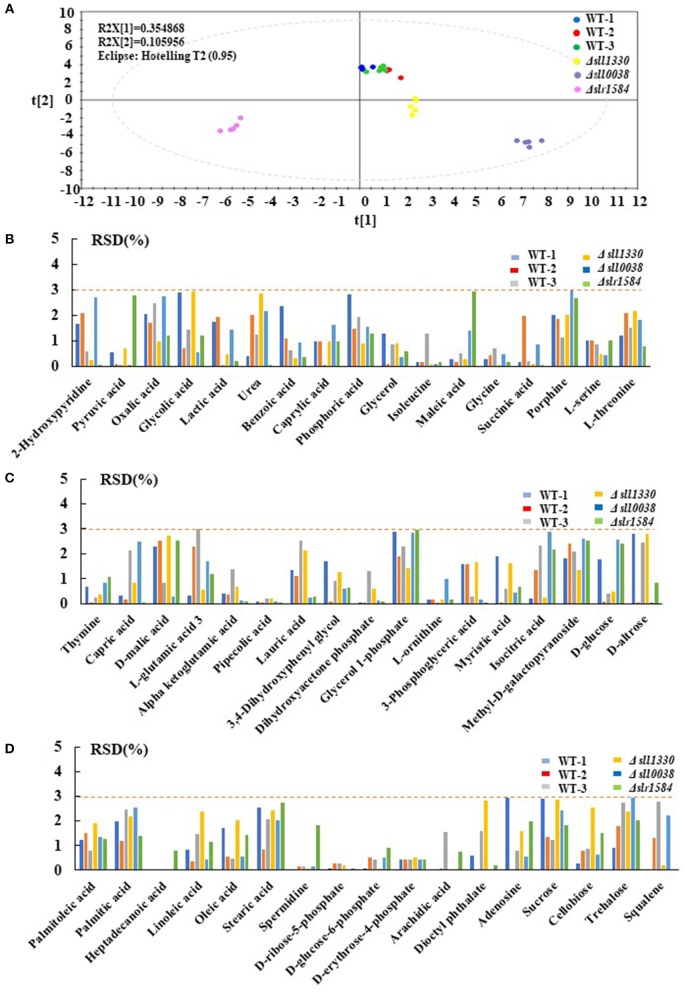
Repeatability analysis three different batches of the wild type and 3 randomly selected mutants. **(A)** PCA plots of 3 different batches wild type and three randomly selected mutants; different colors represent different samples. **(B–D)** RSD analysis of different metabolites detected in the wild type and the mutants; different colors represent different samples.

To demonstrate the reliability of the GC-MS metabolomic analysis, 3 mutants were randomly selected and cultivated under identical autotrophic condition, along with 3 biological replicates of the wild type. Six technical replicates were thus generated for each of the mutant and wild type samples. In the defined analytical conditions, intracellular metabolites were well-separated on the GC column and ~51–70 metabolites were chemically identified among different samples in the MS analysis, with 51 metabolites detected in all samples, including various organic acids, amino acids and sugars identified in practically all samples. The PCA analysis of the metabolic profiles of these samples showed that the technical replicates of each of the mutants or the wild type were clustered well-together in the plot (Figure 1A), suggesting overall good reproducibility of the GC-MS technology employed in this study. In PCA, component 1 and component 2 accounted for 35.49 and 10.60% of the variation, respectively, also suggesting validity and reliability of the method (Wijit et al., 2017). In addition, three biological replicates of the wild type were also closely grouped together, while those of the mutants were well-separated from the wild type and other mutants, demonstrating that not only reproducibility but also sensitivity of the method are in general good (Aranha et al., 2017). Moreover, to demonstrate the measurement accuracy of the 51 metabolites detected in the samples described above, the relative standard deviation (RSD) of all 51 compounds were calculated for all tested samples. The results showed that, among the metabolites identified, the RSD values of most metabolites, such as pipecolic acid, D-glucose-6-phophate, D-ribose-5-phosphate, and D-erythrose-4-phosphate were extremely low around ~0.5%, suggesting the formation of trimethylsilyl derivative (TMS) of these compounds were very reproducible and stable at room temperature and readily to be detected by GC-MS (Lai and Fiehn, 2018). In contrast, several metabolites were detected with RSD values slightly >2.0%, such as phosphoric acid, porphine, glycerol 1-phosphate, isocitric acid, methyl-D-galactopyranoside, palmitic acid and sucrose, implying that these metabolites were relatively unstable in the formation of TMS, which caused larger degree of measurement variation between replicates on GC-MS analysis. Nevertheless, the GC-MS analysis data showed that the RSD values of most 51 compounds were smaller than 3.0% (Figures 1B–D), which is considered acceptable for analysis of semi-quantitation nature (Courant et al., 2013).

**Figure 2:**
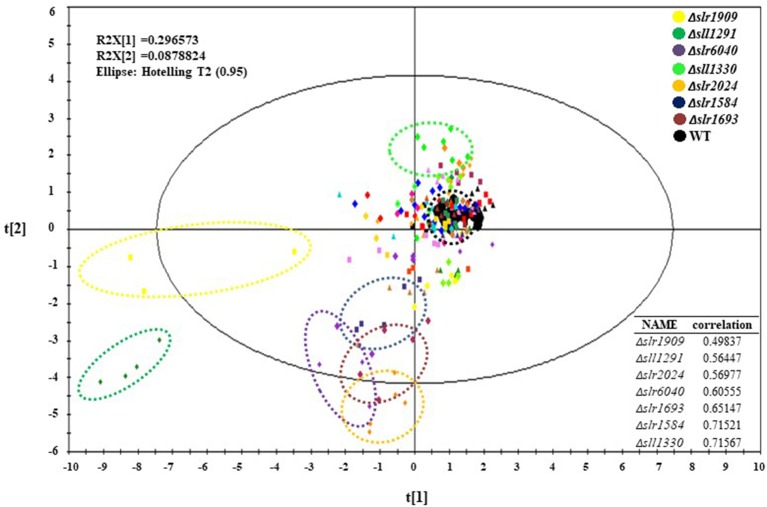
PCA plots of the GC-MS metabolomic profiles at 48 h. Different mutants were represented by different colors.

With the reproducibility and sensitivity of technology demonstrated, the efforts were initiated for analysis of all 44 mutants and the wild type. For the experiments, three biological replicates were prepared for each mutants and wild type samples, each with three technical replicates. Due to the large number of sample cultivation required in this comparative analysis of 44 mutants, mutant samples were cultivated, sampled and analyzed in five batches, each with in parallel cultivation of the wild type control to rectify the possible deviation caused by different cultivation batches. As shown in the PCA plot (Figure 2), the big black dots represent all five batches of the wile type samples, which were clustered well after data normalization, suggesting there was no distinct systematic error caused by experimental arrangement and different batches of cultivation. Interestingly, although the three mutants, Δ*slr0115*, Δ*slr1042*, and Δ*slr1214* showed slightly slow growth patterns under autotrophic condition when compared with the wild type, no dramatic difference of their metabolic profiles from that of the wile type was observed (Figure 2). Meanwhile, the analysis found that seven mutants, namely Δ*slr1909*, Δ*sll1291*, Δ*slr6040*, Δ*sll1330*, Δ*slr2024*, Δ*slr1584*, and Δ*slr1693*, exhibited the most significant changes at the metabolomic level from the wile type, with correlation with the wild type <0.75 (Figure 2), even their growth patterns were almost identical to the wild type (Figure S1), indicating that the knockout of these seven RR genes displayed noteworthy disturbances to the cells at the metabolomic level, suggesting possible regulatory roles of theses RRs in *Synechocystis* grown under autotrophic condition. It worth mentioning that for the mutants without significant difference observed from the PCA plot, it is still possible that the knockout of these RR-encoding genes could cause variations at metabolic level under different cultivation conditions, which we will explore in the future. In the following analysis of this study, we will focus only on the seven mutants in details.

### Integrated Analysis of GC-MS and LC-MS Metabolomic Profiles

**Figure 3:**
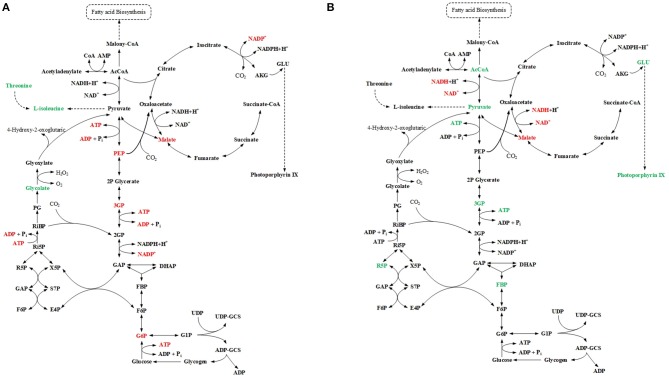
Integrated analysis of GC-MS and LC-MS profiles. **(A)** Pathway view of metabolite changes in Δ*sll1330*, differential regulated metabolites were represented by color, red for down-regulated and green for up-regulated. **(B)** Pathway view of metabolite changes in Δ*slr6040*, differential regulated metabolites were represented by color, red for down-regulated and green for up-regulated.

As the LC-MS metabolomic profiles were also available for the same 44 mutants under the same autotrophic growth condition, an integrated analysis of GC-MS and LC-MS metabolomic profiles was then conducted. Among all 44 mutant, 30 mutants showed no significant changes in both GC-MS and LC-MS metabolomic profiles, suggesting the gene deletion has no visible effects on the metabolites detected under the autotrophic growth condition. Two mutants, Δ*slr1584* and Δ*slr1693*, were found with significant metabolite changes in the GC-MS metabolomic profiles, but without significant variation in the LC-MS metabolomic profiles. Meanwhile, 7 mutants, Δ*sll0797*, Δ*sll0921*, Δ*sll1292*, Δ*slr1042*, Δ*sll1624*, Δ*sll1879*, Δ*slr1588*, displaying obvious changes in the LC-MS metabolomic profiles showed no significant variation in the GC-MS metabolomic profiles compared with the wild type. The results also suggested the merits of using both LC-MS and GC-MS analytic platforms for better coverage of possible changes at metabolite level. Five mutants, Δ*sll1330*, Δ*slr6040*, Δ*slr2024*, Δ*sll1291*, and Δ*slr1909*, showed significant changes identified by both the LC-MS and GC-MS analytic platforms, for which we discussed below:

For mutant Δ*sll1330*, five differentially regulated metabolites were detected by GC-MS analysis, and six differentially regulated metabolites were detected by LC-MS analysis (Figure 3A). Among them D-glucose-6-phosphate was detected in both LC-MS and GC-MS analysis, and was consistently found down-regulated in both profiles. The decreasing amount of NADP, ATP, ADP, 3PG, and PEP observed in LC-MS profiles suggested possible reduction of glycolysis metabolism, which may be relevant to the accumulation of pyruvate and the increasing amount in L-isoleucine, threonine, and D-malic acid in cells. For mutant Δ*slr6040*, three differentially regulated metabolites were detected by GC-MS analysis, and eight differentially regulated metabolites were detected by LC-MS analysis (Figure 3B). The decrease of D-malic acid in TCA circle may be relevant to the decreasing of pyruvate, 3PG, FBP in glycolysis. Similarly, differentially regulated metabolites were identified by both analytical platforms for mutant Δ*slr2024*, Similarly, metabolic pathway views of the changes in Δ*slr2024*, Δ*sll1291*, and Δ*slr1909* were presented in Figures S2–S4, respectively, and available for further analysis.

### Metabolic Changes in Seven Most Regulated RR Mutants

**Figure 4:**
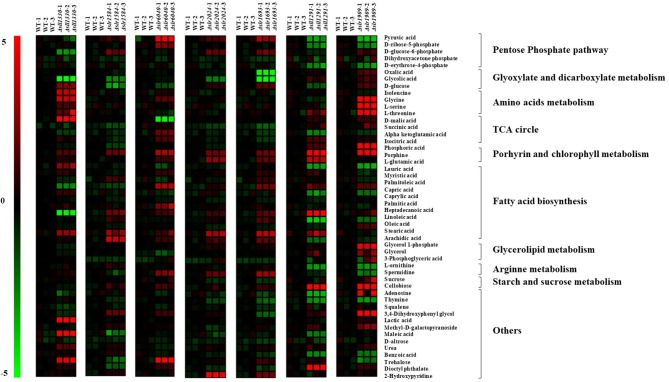
Heat map presentation of the metabolomic changes in 7 most regulated mutants compared with the wild type.

Previous studies showed that Sll1330 involves regulation of the glycolysis genes in *Synechocystis* and the knockout mutant of *sll1330* grew poorly under light-activated heterotrophic growth conditions and overexpression of *sll1330* alters gene expression relevant to the metabolism involved tricarboxylic acid (TCA) cycle and pyruvate metabolism (Tabei et al., 2007; Iijima et al., 2014). In this work, the metabolomic profiling of Δ*sll1330* showed 17 metabolites were with distinctive variation in comparison with the wild type, among which 13 were up-regulated and 4 down-regulated, respectively (Figure 4, Table S2). Notably, intracellular abundance of isoleucine, L-serine, L-threonine, and glycine was increased up to 4-folds, suggesting possible up-regulation of the branched amino acid pathway, and the increase of threonine and serine may also result in the rise of isoleucine as isoleucine was the degradation product of serine and threonine. Like in plants, serine biosynthesis in cyanobacteria involved light-dependent photo-respiratory and light-independent phosphoserine pathways (Klemke et al., 2015). The concentration of urea was also increased 1.67-folds than that of the wild type, in accordance with the up-regulation of several amino acids such as glycine in cyanobacteria (Esteves-Ferreira et al., 2018; Watzer et al., 2019). Three monosaccharides, including D-glucose involved in glycolysis process, methyl-D-galactopyranoside and D-altrose were also up-regulated by more than 2.65-folds respective to the wild type, in line with the possible function of Sll1330 in glycolysis, which was revealed by a previous study that the knockout of *sll1330* could cause the accretion of these monosaccharide (Tabei et al., 2007). Trehalose was increased by 4.40-folds compared with the wild type, its increase is considered to be related to cyanobacterial dehydration tolerance and other stress response (Hershkovitz et al., 1991), suggesting a functional relevance in osmotic tolerance of Sll1330. D-malic involved in TCA cycle was the metabolite increased the most by 7.37-folds than the wild type, suggesting an enhancing activity of the TCA circle, which were consistent with the increased NADPH and decreased phospho(enol)pyruvic acid in Δ*sll1330*, as revealed previously in our LC-MS based analysis (Pei et al., 2017). Four fatty acids were found changed distinctively (e.g. lauric acid, archidic acid, capric acid and linoleic acid), among which the former two were up-regulated nearly 3-folds and the latter two were down-regulated almost 2-folds compared with the wild type. Linoleic acid was unsaturated fatty acid and archidic acid oxidized from unsaturated fatty acid arachidonic acid, which was produced from linoleic acid (Martin et al., 2016). Likewise, lauric acid was produced from capric acid (Sado-Kamdem et al., 2009). D-glucose-6-phosphate involved in both glycolysis and pentose phosphate pathway, was declined nearly 3-folds when compared to the wild type, consistent with our previous analysis using LC-MS which also showed a decreasing of D-glucose-6-phosphate (Pei et al., 2017). Glycolic acid was decreased 6.23-folds than the wild type, which may be due to the possible function of Sll1330 in regulating expression of glycolic acid under light-activated heterotrophic growth conditions (Tabei et al., 2007).

In Δ*slr1584*, there were up to 12 metabolites changed significantly compared with the metabolic profile of the wild type, among which six were up-regulated and six down-regulated, respectively (Figure 4, Table S2). In an early study, the construction and introduction of a gene expression system relevant to green-light regulating from *Synechocystis* 6803 into the cyanobacteria *Synechococcus* sp. NKBG 15041c, resulted in that the expression of *gfp*_*uv*_ as the reporter gene was successfully increased more than 10-folds under green light illumination; while the missing of either the *ccaS* (*sll1473, sll1474, sll1475*) sensor HK genes or the *ccaR* (*slr1584*) RR gene can disrupt the vectors containing *gfp*_*uv*_, thus displayed no detectable induction of GFP_uv_ expression, suggesting *slr1584* gene encodes a cognate RR with photo-responsive capability regulated by green-light (Hirose et al., 2010; Badary et al., 2015). Our metabolomic analysis showed that glycerol-1-phosphate was the most increased metabolite by 4-folds in Δ*slr1584*, which was involved in glycerolipid metabolism and identified as a component of several O-polysaccharides in *E. coli* (Shashkov et al., 2016), and its accumulation suggested possible increased polysaccharides. In addition, lactic acid was increased by 2-folds in Δ*slr1584* mutant, suggesting a possible relevance of *sll1584* function in lactate metabolism (Sanchez et al., 1975). D-glucose was the metabolite down-regulated the most by 3.05-folds. Succinic acid involved in TCA circle was also found decreased by 1.53-folds, in consistent with our LC-MS previous result that both α-ketoglutaric acid and oxaloacetic acid of TCA cycle intermediates were declined in Δ*slr1584* (Pei et al., 2017).

Slr6040 was a plasmid pSYSX-encoded RR in *Synechocystis*, and a previous study showed that expression of *rre*P (*slr6040*) was changed significantly over time in nitrogen-depleted Δ*glgC* mutant respective to the wild type (Carrieri et al., 2017). In Δ*slr6040*, our metabolomic analysis showed that up to 15 metabolites were significantly regulated in comparison with the that of the wild type, among which 12 were up-regulated and 3 down-regulated (Figure 4, Table S2). With the highest fold increase of 5.13, the accumulation of trehalose indicated a possible relevance to osmotic response of the RR Slr6040. In addition, pyruvic acid was increased by 4.83-folds. In addition, α-ketoglutaric and D-ribose-5-phosphate were indeed increased by 2.16- and 2.58-folds, respectively, also consistent with our previous LC-MS analysis of the same mutant (Pei et al., 2017). D-malic acid was decreased the most by nearly 5-folds, suggesting a possible involvement of *slr6040* in regulating TCA cycle (Yin et al., 2015).

Slr2024, a RR belonged to the CheY family, was previously found associated with thylakoid membranes by electroblotting and *N*-terminal sequencing (Wang et al., 2000) and was potentially involved in the redox signaling linked to the photochemistry, as *slr2024* was down-regulated in response to high light (Singh et al., 2008). Compared with the metabolic profile of the wild type, 9 metabolites were found changed significantly in Δ*slr2024*, among which 8 were up-regulated and 1 down-regulated (Figure 4, Table S2). 2-hydroxypyridine is the most increased metabolite by 4.68-folds. In addition, glycolic acid was down-regulated by 2.50-folds, implying possible regulatory roles of *slr2024* in glyoxylate and diglyoxylate metabolism (Kisaki and Tolbert, 1969), in consistence with decrease of D-fructose 6-phosphate and D-fructose 1,6-bisphosphate under autotrophic condition but increase under light-activated heterotrophic condition, as revealed in our previous LC-MS analysis of the Δ*slr2024* mutant (Pei et al., 2017).

Previous studies showed that Slr1694 (PixD) is a blue light receptor with a BLUF (blue light sensors by a flavin chromophore) domain and the protein-protein interaction between PixD and a PatA-like RR PixE (Slr1693) is a requisite to accomplish light signal transduction process of phototaxis in *Synechocystis* (Tanaka et al., 2012). In Δ*slr1693*, 14 metabolites were found displaying variation in the metabolic profile compared with that of the wild type, among which nine were up-regulated and five down-regulated (Figure 4, Table S2). As the saturated formation of eicosenoic acid (20:1Δ^5^) (Cahoon et al., 2000), arachidic acid (20:0) was increased the most by 3.76-folds, indicating a relevance of the *slr1693* gene in fatty acid synthesis. In addition, sucrose that was increased by 3.07-folds, could work as a compatible solute to cope with salinity fluctuations and maintain the intracellular osmotic balance (Kolman and Salerno, 2016) and its declination indicated a potential response of *slr1693* in salt tolerance. Moreover, glycolic acid and oxalic acid were down-regulated by 8.07- and 4.91-folds, respectively, in consistent with the decreased abundance of NADPH and oxaloacetic acid in the Δ*slr1693* mutant, as revealed in our previous LC-MS analysis of the same mutant (Pei et al., 2017).

A previous study showed that *sll1291* gene was expressed at a lower level in the *isaR1* (Iron-Stress-Activated small RNA 1) overexpressing strain, suggesting its regulatory roles in iron stress since IsaR1 plays a pivotal role in acclimation to low-iron conditions (Georg et al., 2017). In addition, studies also found that *sll1291* was part of a gene cluster involved in regulating chemotaxis (Singh et al., 2010) and circadian rhythm in *Synechocystis* (Kucho et al., 2005). Moreover, comparative genomics analysis of NtcA predicted that the *sll1291* was part of the NtcA regulon (Su et al., 2005), and sugar metabolism was motivated by the control of NtcA during nitrogen depletion thus *sll1330* and *sll1291* were actually induced by nitrogen depletion (Osanai et al., 2006). In this study, the metabolic profiling of Δ*sll1291* showed that 22 metabolites were differentially regulated compared with the wild type, among which 8 were up-regulated and 14 down-regulated (Figure 4, Table S2). Porphine was increased the most by 19.78-folds. Additionally, the amount of adenosine was up-regulated by nearly 6-folds, also in consistent with the increased intracellular abundance of ADP-glucose, ATP and ADP in the Δ*sll1291*, as revealed in our previous LC-MS analysis (Pei et al., 2017). Moreover, with linoleic acid increased by 8.40-folds and oleic acid decreased by 3.98-folds, we speculated that *sll1291* might be involved in the regulation of the metabolism of linoleic acid. α-ketoglutamic acid was decreased by 2.62-folds in the Δ*sll1291* mutant, which probably due to the fact that *sll1291* was part of the NtcA regulon (Su et al., 2005), and cellular abundance of 2-oxoglutarate was influenced by the nitrogen source (Herrero et al., 2001).

Comparative growth analysis showed that Δ*slr1909* grew poorly in BG11 medium at pH 6.2–6.5 in comparison with the wild type and further analysis showed that Slr1909 was relevant to the response to acidic environment (Ren et al., 2014). Our metabolomic analysis showed that 24 metabolites were displaying distinct variances in Δ*slr1909* in comparison with the wild type, among which 15 were up-regulated and 9 down-regulated (Figure 4, Table S2). The metabolite with the highest increased folds was L-serine by more than 15-folds. In the absence of a complete glycolate pathway in cyanobacteria, the synthesis of serine directly from phosphoglycerate (PGA) was therefore necessary to ensure a consistent supply of this amino acid for protein synthesis, which was compatible with the increasing of 3-phosphoglyceric acid (Colman and Norman, 1997). The proliferation of serine indicated a potential connection of *slr1909* in the phosphoserine pathways. Additionally, glycine and threonine that are related to serine in the metabolic pathway, were increased by 14.80- and 10.85-folds, respectively. It worth noting that porphine also showed a high increasing fold similar to that we found with Δ*sll1291*, which may worth further investigation whether the two RR genes are functional related. Additionally, pyruvic acid, D-erythrose-4-phosphate and D-ribose-5-phosphate were declined 3.76-, 2.91-, and 2.72-folds, respectively, among which pyruvic is a crucial intermediate in glycolysis and the latter two are key components of oxidative pentose phosphate pathway and Calvin circle, implicating a possible relevance of the Sll1909 in sugar metabolism, which was also in accordance with the increasing amount of ADP-glucose, AMP, D-Glucose 6-phosphate, D-Fructose 6-phosphate, D-Ribose 5-phosphate, and DL-Glyceraldehyde 3-phosphate in the Δ*slr1909* mutant under photo-mixotrophic condition previously revealed by LC-MS based analysis (Pei et al., 2017).

### RT-qPCR Confirmation of the Metabolic Changes

**Figure 5:**
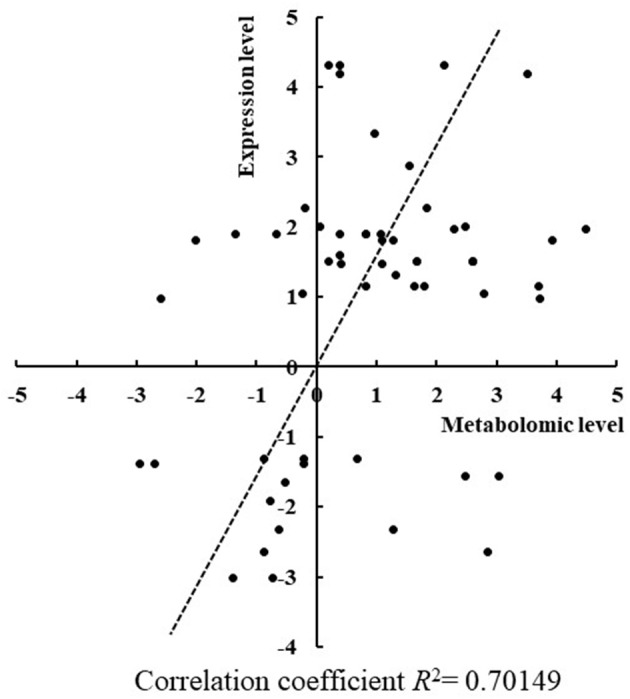
Correlation between changes of gene expression level and metabolomics level in mutants. The changes of gene expression level and metabolomics level showed a good correlation since *R*^2^ = 0.70.

To confirm some of the findings we obtained from the comparative metabolomic analysis between the RR mutants and the wild type, RT-qPCR analysis was employed to determine expression changes of key genes involved in the differentially regulated metabolic pathways in the seven mutants. Toward the goal, differentially regulated metabolites identified by GC-MS analysis were first mapped into the metabolic pathways according to the KEGG analysis to determine the responsive metabolic pathways in each mutant, and then at least three genes were selected from the differentially regulated metabolic pathways and their expression were comparatively determined in the mutants. In total, expression of 27 genes involved in production and consumption of the differentially regulated metabolites were comparatively determined between the mutants and the wild type. As summarized in Table 1, in general good correlation was obtained for a majority of genes where the similar regulation patterns were observed in both metabolomic analysis and RT-qPCR analysis (Figure 5). In addition, we also found that the variations of metabolites were possibly resulted from the co-functioning of genes involved in production and consumption. For example, in Δ*sll1330*, genes *slr0009* (*rbcL*), and *slr0012* (*rbcS*) involved in the production of glycolic acid were down-regulated 1.83- and 3.30-folds, respectively, as well as the gene *sll1349* (*cbbZp*) involved in consumption of glycolic acid was up-regulated 7.23-folds, which together may lead to the decrease of glycolic acid. Meanwhile, the production gene *slr1349* (*pgi*) of D-glucose-6-phosphate was up-regulated for 5.59-folds and the consumption gene *slr1843* (*zwf*) up-regulated for 8.16-folds, respectively, which may lead to the down-regulation of D-glucose-6-phosphate for 2.97-folds in the metabolomics profile. The similar observation also applied to Δ*slr2024* regarding genes involved in production and consumption of L-glutamic acid. In Δ*slr1909*, pyruvic acid was down-regulated 3.76-folds, which may be resulted from the combined effects of the down-regulated *slr0721* (*me*) and up-regulated *sll1721* (*pdhB*).

**Table 1:** RT-qPCR confirmation of the metabolic changes in seven most regulated RR mutants.

**Mutants**	**Responsive metabolites**	Metabolomic_fold change^*^	**Production gene ID**	**Function and pathways**	RT-PCR_fold change^**^	**Consumption Gene ID**	**Function and Pathways**	RT-PCR_fold change^**^
Δ*sll1330*	D-malic acid	7.37	*sll0891*	*citH*, encoding a malate dehydrogenase	2.91	*sll0018*	*fumC*, fumarate hydratase	1.83
	Glycolic acid	−6.23	*slr0009*	*rbcL*, ribulose bisphosphate carboxylase, RuBisCO large subunit	−1.83	*sll1349*	*cbbZp*, phosphoglycolate phosphatase	7.23
			*slr0012*	*rbcS*, ribulose bisphosphate carboxylase, RuBisCO small subunit	−3.30			
	Isoleucine	3.89	*slr0032*	*ilvE*, encoding branched-chain amino acid aminotransferase	22.23	*slr1096*	*phdD*, dihydrolipoamide dehydrogenase	4.92
	L-threonine	2.79	*sll1172*	*thrC*, threonine synthase	2.14	*slr2072*	*ilvA*, encoding a L-threonine deaminase	1.33
	D-glucose-6-phosphate	−2.97	*slr1349*	*pgi*, glucose-6-phosphate isomerase	5.59	*slr1843*	*zwf*, glucose-6-phosphate 1-dehydrogenase	8.16
Δ*slr1584*	Glycerol 1-phosphate	4.00	*sll1973*	involved in the glycerophospholipid metabolism	5.56	*sll1848*		1.04
							1-acyl-sn-glycerol-3-phosphate acyltransferase	
	D-glucose-6-phosphate	2.07	*slr1349*	*pgi*, glucose-7-phosphate isomerase	6.91	*slr1843*	*zwf*, glucose-6-phosphate 1-dehydrogenase	−1.17
	Glycolic acid	−2.60	*slr0009*	*rbcL*, ribulose bisphosphate carboxylase, RuBisCO large subunit	−6.51	*sll1349*	*cbbZp*, phosphoglycolate phosphatase	1.47
			*slr0012*	*rbcS*, ribulose bisphosphate carboxylase, RuBisCO small subunit	−7.71			
Δ*slr6040*	D-malic acid	−4.99	*sll0891*	*citH*, encoding a malate dehydrogenase	−1.56	*sll0018*	*fumC*, fumarate hydratase	2.44
	Pyruvic acid	4.83	*slr0721*	*me*, encoding a malic enzyme, involved in pyruvate metabolism	3.58	*sll1721*	*pdhB*, pyruvate dehydrogenase E1 component beta subunit	−1.14
	Porphine	3.49	*slr0839*	*hemH*, chlorophyll biosynthesis	15.16	*sll1899*	*ctaB*, cytochrome c oxidase folding protein	−4.02
			*slr1030*	*chlI*, chlorophyll biosynthesis	2.42			
			*slr0525*	*chlM*, chlorophyll biosynthesis	2.13			
Δ*slr2024*	D-glucose-6-phosphate	1.96	*slr1349*	*pgi*, glucose-6-phosphate isomerase	−6.04	*slr1843*	*zwf*, glucose-6-phosphate 1-dehydrogenase	7.74
	Glycolic acid	−2.5	*slr0009*	*rbcL*, ribulose bisphosphate carboxylase, RuBisCO large subunit	−1.83	*sll1349*	*cbbZp*, phosphoglycolate phosphatase	−1.16
			*slr0012*	*rbcS*, ribulose bisphosphate carboxylase, RuBisCO small subunit	−1.16			
	Porphine	2.21	*slr0839*	*hemH*, chlorophyll biosynthesis	3.46	*sll1899*	*ctaB*, cytochrome c oxidase folding protein	1.77
			*slr1030*	*chlI*, chlorophyll biosynthesis	3.09			
			*slr0525*	*chlM*, chlorophyll biosynthesis	12.95			
	L-glutamic acid	2.86	*slr0288*	*glnN*, glutamine synthetase	1.14	*sll1027*	*gltD*, glutamate metabolism	3.17
			*slr1756*	*glnA*, glutamine synthetase	1.31	*sll1499*	*gltB*, glutamate metabolism	6.11
			*sll1502*	*gltB*, glutamate metabolism	3.17	*sll1641*	*gad*, glutamate decarboxylase	−8.98
Δ*slr1693*	Glycolic acid	−8.07	*slr0009*	*rbcL*, ribulose bisphosphate carboxylase, RuBisCO large subunit	−2.63	*sll1349*	*cbbZp*, phosphoglycolate phosphatase	1.60
			*slr0012*	*rbcS*, ribulose bisphosphate carboxylase, RuBisCO small subunit	−1.66			
	Pyruvic acid	3.01	*slr0721*	*me*, encoding a malic enzyme, involved in pyruvate metabolism	1.32			
Δ*sll1291*	Porphine	19.78	*slr0839*	*hemH*, chlorophyll biosynthesis	1.30			
			*slr1030*	*chlI*, chlorophyll biosynthesis	1.15			
			*slr0525*	*chlM*, chlorophyll biosynthesis	4.36			
	L-glutamic acid	3.74	*slr0288*	*glnN*, glutamine synthetase	2.11	*sll1027*	*gltD*, glutamate metabolism	−1.58
			*slr1756*	*glnA*, glutamine synthetase	6.07	*sll1499*	*gltB*, glutamate metabolism	1.77
			*sll1502*	*gltB*, glutamate metabolism	−2.57	*sll1641*	*gad*, glutamate decarboxylase	−1.50
	Pyruvic acid	−3.15	*slr0721*	*me*, encoding a malic enzyme, involved in pyruvate metabolism	−1.44			
Δ*slr1909*	L-threonine	18.28	*sll1172*	*thrC*, threonine synthase	11.45	*slr2072*	*ilvA, encoding a L-threonine deaminase*	1.30
	Phosphoric acid	10.15	*slr1124*	*gpmB*, phosphoserine phosphatase, involved in glycine, serine and threonine metabolism	1.96	*sll1908*	*serA*, D-3-phosphoglycerate dehydrogenase / 2-oxoglutarate reductase	−1.73
	Pyruvic acid	−3.76	*slr0721*	*me*, encoding a malic enzyme, involved in pyruvate metabolism	−1.70	*sll1721*	*pdhB*, pyruvate dehydrogenase E1 component beta subunit	2.30
	D-glucose-6-phosphate	2.47	*slr1349*	*pgi*, glucose-6-phosphate isomerase	2.49	*slr1843*	*zwf*, glucose-6-phosphate 1-dehydrogenase	−1.95

* *Metabolomic_fold change represents the changes of metabolomic level compared with the wild type*.

** *RT-PCR_fold change represents the gene expression level compared with the wild type*.

### Comparative Analysis of Metabolomic and Evolutionary Profiles

**Figure 6:**
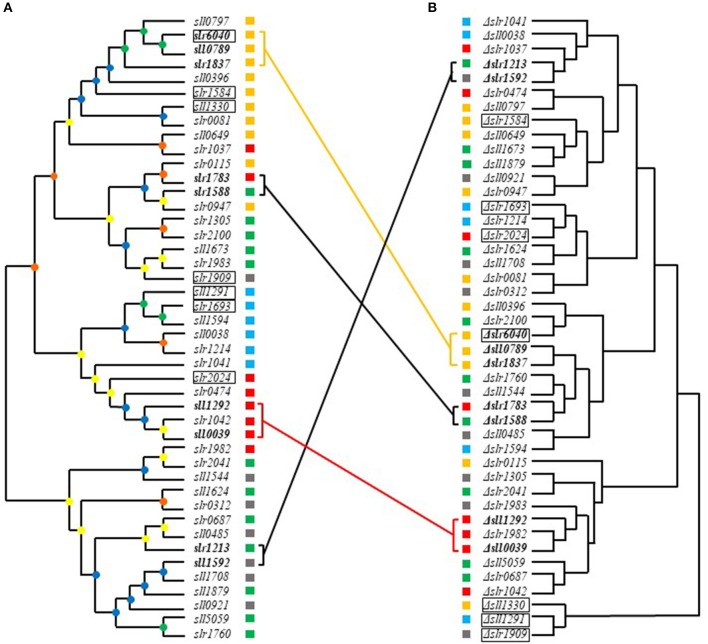
Comparative analysis of metabolomics- and evolution-based trees of RRs. **(A)** Phylogenetic analysis of RRs based on the receiver domains. Different cube colors beside the gene names and mutants represent different subfamilies: gray for NarL family, yellow for OmpR family, red for CheY family, blue for PatA family and green for others, respectively. **(B)** Hierarchical clustering analysis based on metabolomic profiles. Different cube colors beside the gene names and mutants represent different subfamilies: gray for NarL family, yellow for OmpR family, red for CheY family, blue for PatA family and green for others, respectively. Four groups of RR genes that shared close relationship in both phylogenetic tree and metabolomics-based tree were marked in bold. Genes clustered in both metabolomics- and evolution-based trees were linked by lines with same colors of their subfamilies. The seven most regulated mutants were marked with black frame. The bootstrap values were represented in different colors of nodes: green for 1,000–750, blue for 750–500, yellow for 500–250, and orange for 250–0.

Hierarchical clustering analysis of metabolomic profiles was further performed. In addition, a phylogenetic tree based on the conserved receiver domains of the 44 RR genes was also constructed (Figure 6A). A comparison between trees constructed from metabolomic data and evolutionary relationship was then conducted (Figure 6). It was previously reported that most of RRs in *Synechocystis* can be grouped into a few subfamilies with output domains involved in corresponding regulatory roles, namely OmpR, NarL, CheY, and PatA subfamilies (Mizuno et al., 1996). Consistently, such clustering patterns were also observed in the phylogenetic tree we constructed using the conserved receiver domains of the 44 RR genes (Figure 6A).

Comparative metabolomic and evolutionary analysis first allowed identification of four groups of RR genes that not only clustered well in phylogenetic trees but also in metabolomic clustering trees, including *slr6040, sll0789*, and *slr1837, slr1588* and *slr1783, sll1292* and *sll0039, sll1592* and *slr1213*, suggesting possible functional conservation of these RRs during evolution (Figure 6).

In group one, *slr6040* was found clustered with *sll0789* and *slr1837* in both metabolomics- and evolution-based trees. All belong to the OmpR subfamily. Slr1837 (ManR) functioning with Slr0640 (ManS), has been previously revealed to regulate manganese homeostasis in *Synechocystis* (Ogawa et al., 2002). The expression of RR-HK pair Sll0789–Sll0790, which was adjacent to the gene cluster of metal resistance, downstream of *ziaA* in the *Synechocystis* genome, were declined in the Δ*sll0649* mutant under Cd^2+^ stress (Chen et al., 2014b). While the *sll0788* upstream of *sll0789* and *sll0790*, along with *slr6039, slr6040*, and *slr6041* in endogenous plasmids pSYSX in *Synechocystis*, were both essential for copper resistance (Giner-Lamia et al., 2012). Interesting, our metabolomic analysis showed that D-glucose and heptadecanoic acid were regulated similarly in these 3 knockout mutants Δ*sll0789*, Δ*slr6040*, and Δ*slr1837*, with the D-glucose declined more than 1.5-folds and heptadecanoic acid enhanced more than 1.5-folds, suggesting these three RR genes may function on the similar metabolism in cells.

In group two, the *sll1588* gene shared high similarity in both metabolomics- and evolution-based trees with *slr1783* of NarL subfamily (Figure 6). Slr1783 (Rre1) has previously been demonstrated to interact with Hik2 using a yeast two-hybrid analysis of cyanobacterial TCSTSs (Sato et al., 2007) and Hik2 was involved in a signaling pathway required in accommodation to light and salinity in cyanobacteria (Ibrahim et al., 2016), while Slr1588 was also shown relevant to salt tolerance in our previous study (Chen et al., 2014a). The metabolites decreased more than 1.5-folds in both of Δ*slr1588* and Δ*slr1783* included oxalic acid, phosphoric acid, D-glucose, D-ribose-5-phosphate, D-glucose-6-phosphate, which are related to the glycolytic pathway. Meanwhile, metabolites L-serine, 3-phosphoglyceric acid and heptadecanoic acid were increased more than 1.5-folds in both mutants, suggesting roles of *slr1588* and *slr1783* in branched amino acids pathway and fatty acids metabolism.

In group three, *sll1292* and *sll0039* both belong to CheY subfamily. In a previous study, both Slr1982 and Sll0039 were identified as proteins relevant to butanol stress (Tian et al., 2013). The co-overexpression of *sll0039* and *slr1037* relative to the butanol tolerance of *Synechocystis* (Gao et al., 2017) improves butanol tolerance nearly by 1.3-folds (Niu et al., 2015). With an early study showed that *sll1292* was responsive to the deficiency of inorganic carbon (Wang et al., 2004), Sll1292 was also found down-regulated under 0.8% (v/v) hexane stress (Liu et al., 2012). Metabolites pyruvic acid, glycolic acid, maleic acid, D-malic acid, and linoleic acid were declined nearly 1.5-folds in both mutants of Δ*sll1292* and Δ*sll0039*, while isocitric acid was increased more than 2-folds, suggesting the possible relevance to the TCA circle in *Synechocystis*.

In group four, *sll1592* and *slr1213*, with the former belonged to NarL subfamily and the latter with no specific subfamily association also displayed similarity in both metabolomics- and evolution-based trees. The proteins Sll1590 (Hik20) and Sll1592 (Rre19) of *Synechocystis* are corresponding homologous to KdpD and KdpE of *E. coli*, respectively, which has been identified as the second TCSTS related to osmotic signals in *E. coli* (Paithoonrangsarid et al., 2004). *slr1213* (*uirR*) and *slr1214* (*lsiR*) encode a UVA-activated signaling system which was previously found essential in phototaxis (Song et al., 2011). No evidence of their similar function was previously available. In Δ*sll1592* and Δ*slr1213*, the metabolite L-(+) lactic acid was increased more than 1.5-folds while D-malic acid, D-glucose and D-ribose-5-phosphate were declined more than 1.5-folds, the variations of the latter three metabolites indicated relevant role of the genes to TCA circle, glycolysis, Calvin circle, and pentose phosphate pathway, respectively.

In addition, more than 10 groups of RR genes that were clustered together in the evolution tree were found far away from each other in metabolomics-based tree (Figure 6). For example, the OmpR family genes *slr1584* and *sll1330* were different in terms of their metabolomic profiles, suggesting clearly different regulatory function and possible function change during the evolution of *Synechocystis*.

Moreover, six groups of RR genes that were clustered in metabolomics-based tree but well-separated with each other in evolution-based tree, suggesting different evolutionary origin, such as *slr1909* and *sll1291, slr1214*, and *slr2024*. Although *slr1909* and *sll1291* belong to different clusters in evolutionary tree (*slr1909* belongs to NarL and *sll1291* to PatA subfamilies, respectively), metabolomic clustering analysis showed they were well-clustered together (Figure 6B), suggesting either a possible functional convergence during evolution or they were both involved in the same regulatory pathway, which worth further investigation. As a part of the NtcA regulon (Su et al., 2005), Sll1291 was previously found involved in the regulation of response to iron stress (Georg et al., 2017), while a previous study showed that *slr1909* was relevant to acid response (Ren et al., 2014). In addition, *sll1291* was found to be part of a gene cluster involved in chemotaxis (Singh et al., 2010). Also, as the important element of chlorophyll, porphine was increased more than 10-folds in both Δ*slr1909* and Δ*sll1291* mutants, which also suggested a high relevance in phototactic motility of the two genes.

The metabolomic profiling analysis showed that *slr2024* of CheY subfamily was clustered with *slr1214* of PatA subfamily in the metabolomics clustering tree, but slightly distant from *slr1214* in the evolutionary trees. Slr1214 was involved in a UVA-activated signaling system that is required for negative phototaxis (Song et al., 2011), while Slr2024 was found associated with thylakoid membranes (Wang et al., 2000), and down-regulated in responsive to high light (Singh et al., 2008). The common relevance in light response may explain the high functional similarity of *slr1214* and *slr2024* in metabolomic profiles. Metabolites pyruvic acid and porphine were increased more than 1.5-folds while glycolic acid was decreased more than 1.5-folds in Δ*slr1214* and Δ*slr2024*.

## Conclusion

In this study, a GC-MS based comparative metabolomic analysis was conducted for a library of knockout mutants for 44 RRs in *Synechocystis*. The metabolomic profiling showed that seven RRs mutants were significantly different at metabolomic level while no distinctive variance in growth was observed under the normal autotrophic growth condition, suggesting diversity of regulation mediated by RRs in *Synechocystis*. Additionally, an integrative metabolomic and evolutionary analysis of all RR mutants led to the identification of four groups of RR genes which shared close relationship in both phylogenetic and metabolomics-based trees, suggesting possible function conservations of these RRs during the evolutionary process. Meanwhile, six groups of RRs with close evolutionary origin were found with different metabolomic profiles, suggesting possible functional changes during evolution. In contrast, more than 10 groups of RR genes with different clustering patterns in the evolutionary tree were found clustered together in metabolomics-based tree, suggesting possible functional convergences during the evolution. This study provided new insights into the regulation diversity and the evolution of TCSTS in *Synechocystis*.

## Data Availability Statement

All datasets generated for this study are included in the article/Supplementary Material.

## Author Contributions

MS conducts experiments and analyzes the data and writes the manuscript. LC analyzes the data and writes the manuscript. WZ designs the experiments and writes the manuscript.

### Conflict of Interest

The authors declare that the research was conducted in the absence of any commercial or financial relationships that could be construed as a potential conflict of interest.

## Supplementary Material

Figure S1 Growth curves for 7 most-regulated mutants compared with the wile type.

Figure S2 Pathway view of metabolite changes in Δ*slr2024*, differential regulated metabolites were represented by color, red for down-regulated and green for up-regulated metabolites.

Figure S3 Pathway view of metabolite changes in Δ*sll1291*, differential regulated metabolites were represented by color, red for down-regulated and green for up-regulated metabolites.

Figure S4 Pathway view of metabolite changes in Δ*slr1909*, differential regulated metabolites were represented by color, red for down-regulated and green for up-regulated metabolites.

Table S1 Primers used for quantitative real-time RT-PCR in this study.

Table S2 GC-MS metabolomics dataset of 44 mutants under autotrophic condition.

## Abbreviations

GC-MS: gas chromatography-mass spectrometry
HK: histidine kinase
LC-MS: liquid chromatography-mass spectrometry
PCA: principal component analysis
PGA: phosphoglycerate
RR: receiver response regulator
RSD: relative standard deviation
RT-qPCR: real-time quantitative polymerase chain reaction
TCA: tricarboxylic acid cycle
TCSTSs: Two-component signal transduction systems.
