# Supplementary figures and images for: Regulatory Diversity and Functional Analysis of Two-Component Systems in Cyanobacterium Synechocystis sp. PCC 6803 by GC-MS Based Metabolomics
Source: Front Microbiol. 2020 Mar 17;11:403. doi: 10.3389/fmicb.2020.00403 (PMC7090099; doi:10.3389/fmicb.2020.00403)

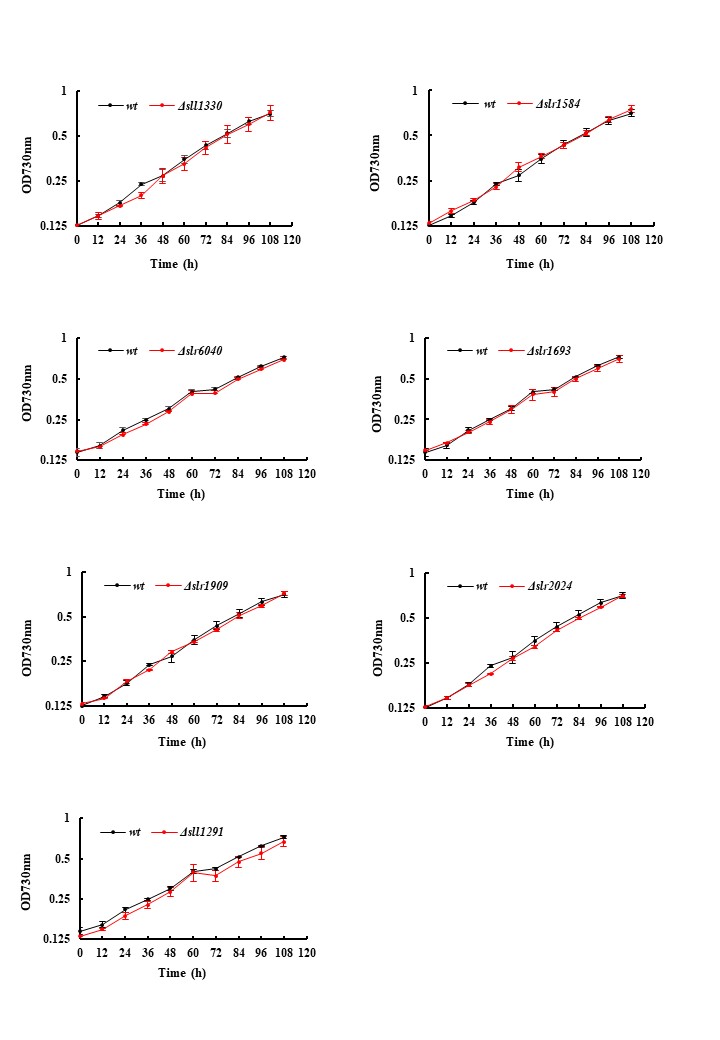

Supplement: Figure S1 — Growth curves for 7 most-regulated mutants compared with the wile type. [file Image_1.JPEG]

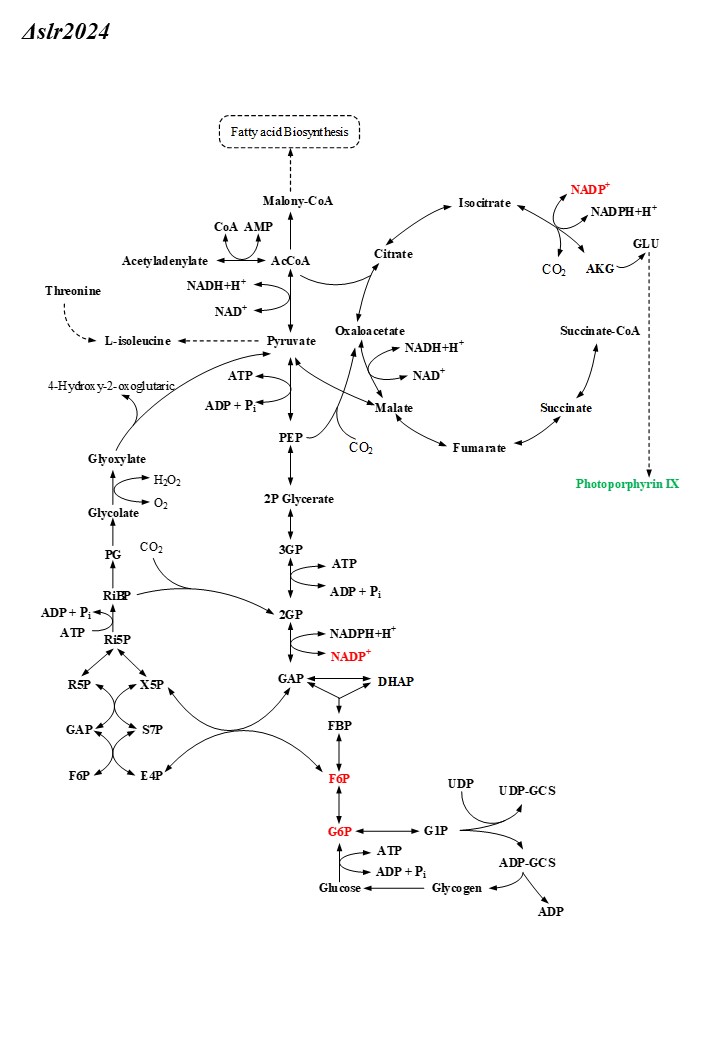

Supplement: Figure S2 — Pathway view of metabolite changes in Δslr2024, differential regulated metabolites were represented by color, red for down-regulated and green for up-regulated metabolites. [file Image_2.JPEG]

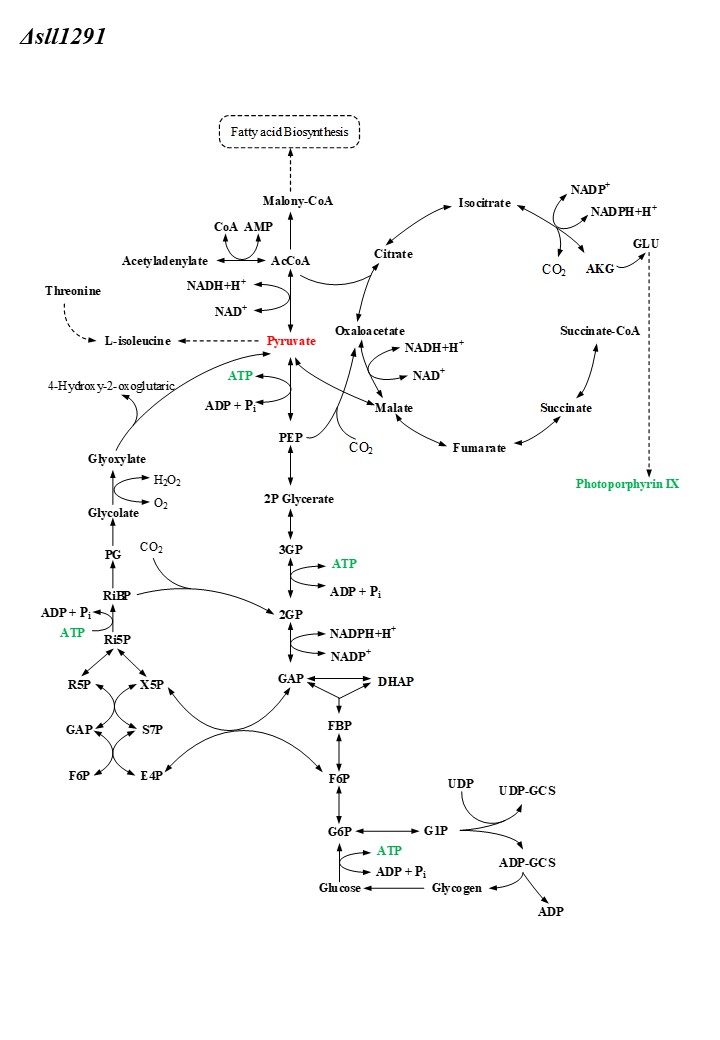

Supplement: Figure S3 — Pathway view of metabolite changes in Δsll1291, differential regulated metabolites were represented by color, red for down-regulated and green for up-regulated metabolites. [file Image_3.JPEG]

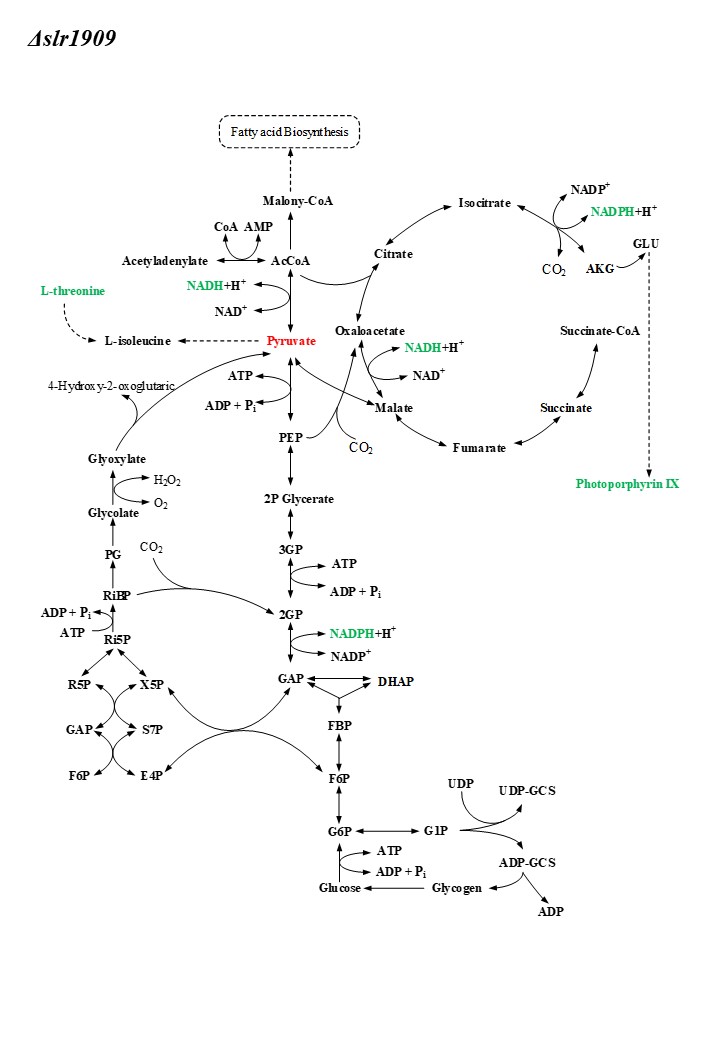

Supplement: Figure S4 — Pathway view of metabolite changes in Δslr1909, differential regulated metabolites were represented by color, red for down-regulated and green for up-regulated metabolites. [file Image_4.JPEG]
